# miRNA-126-3p carried by human umbilical cord mesenchymal stem cell enhances endothelial function through exosome-mediated mechanisms in vitro and attenuates vein graft neointimal formation in vivo

**DOI:** 10.1186/s13287-020-01978-z

**Published:** 2020-11-02

**Authors:** Qingxi Qu, Limei Wang, Weidong Bing, Yanwen Bi, Chunmei Zhang, Xuanxuan Jing, Linghong Liu

**Affiliations:** 1 Department of Obstetrics and Gynecology, Qilu Hospital of Shandong University, Jinan, 250012 People’s Republic of China; 2 Department of Cardiovascular Surgery, Qilu Hospital of Shandong University, Jinan, 250012 People’s Republic of China; 3 Department of Cardiology, Qilu Hospital of Shandong University, Jinan, 250012 Shandong People’s Republic of China; 4 Department of Ultrasound, Qilu Hospital of Shandong University, Jinan, 250012 Shandong People’s Republic of China; 5 Laboratory of Cryomedicine, Qilu Hospital of Shandong University, Jinan, 250012 Shandong People’s Republic of China; 6 Research Center of Stem Cell and Regenerative Medicine, Shandong University, Jinan, 250012 Shandong People’s Republic of China

**Keywords:** miRNA-126-3p, Exosomes, Mesenchymal stem cell, Vein graft, Reendothelialization, Neointimal hyperplasia

## Abstract

**Background:**

The aim of this study was to determine whether the combination of MSC implantation with miRNA-126-3p overexpression would further improve the surgical results after vein grafting.

**Methods:**

human umbilical cord MSCs (hucMSCs) and human umbilical vein endothelial cells (HUVECs) were isolated from human umbilical cords and characterized by a series of experiments. Lentivirus vector encoding miRNA-126-3p was transfected into hucMSCs and verified by PCR. We analyzed the miRNA-126-3p-hucMSC function in vascular endothelial cells by using a series of co-culture experiments. miRNA-126-3p-hucMSCs-exosomes were separated from cell culture supernatants and identified by WB and TEM. We validated the role of miRNA-126-3p-hucMSCs-exosomes on HUVECs proliferative and migratory and angiogenic activities by using a series of function experiments. We further performed co-culture experiments to detect downstream target genes and signaling pathways of miRNA-126-3p-hucMSCs in HUVECs. We established a rat vein grafting model, CM-Dil-labeled hucMSCs were injected intravenously into rats, and the transplanted cells homing to the vein grafts were detected by fluorescent microscopy. We performed historical and immunohistochemical experiments to exam miRNA-126-3p-hucMSC transplantation on vein graft neointimal formation and reendothelialization in vitro.

**Results:**

We successfully isolated and identified primary hucMSCs and HUVECs. Primary hucMSCs were transfected with lentiviral vectors carrying miRNA-126-3p at a MOI 75. Co-culture studies indicated that overexpression of miRNA-126-3p in hucMSCs enhanced HUVECs proliferation, migration, and tube formation in vivo. We successfully separated hucMSCs-exosomes and found that miRNA-126-3p-hucMSCs-exosomes can strengthen the proliferative, migratory, and tube formation capacities of HUVECs. Further PCR and WB analysis indicated that, SPRED-1/PIK3R2/AKT/ERK1/2 pathways are involved in this process. In the rat vein arterialization model, reendothelialization analysis showed that transplantation with hucMSCs modified with miRNA-126-3p had a higher reendothelialization of the vein grafts. The subsequent historical and immunohistochemical examination revealed that delivery with miRNA-126-3p overexpressed hucMSCs significantly reduced vein graft intimal hyperplasia in rats.

**Conclusion:**

These results suggest hucMSC-based miRNA-126-3p gene therapy may be a novel option for the treatment of vein graft disease after CABG.

## Introduction

Coronary atherosclerotic heart disease (CHD) is considered one of the most serious diseases threatening human life and health. Coronary artery bypass grafting (CABG) has become the main treatment for severe cases of CHD. For CABG, the autogenous great saphenous vein is the most widely used vascular graft [1]. However, more than half of vein grafts will be occluded during the first 10 years after CABG [2]. The restenosis caused by vein graft intimal hyperplasia has become a key factor influencing the efficacy of CABG. Therefore, a method to increase long-term patency rate of the autogenous vein graft has become a hot topic of research [3].

The initiation and trigger of vein graft intimal hyperplasia is the damage and loss of endothelial integrity caused by operative injury, hemodynamic changes, and inflammation [4]. The main pathology of vein graft restenosis highlights the abnormal proliferation of subendothelial smooth muscle cells and increased deposition of extracellular matrix [5]. Thus, repairing the damaged endothelium and improving endothelial function to accelerate reendothelialization is crucially important to prevent the initiation and development of vein graft intimal thickening and failure.

MSC-mediated therapies hold great promise for tissue repair and regeneration, especially in vascular diseases, such as pulmonary arterial hypertension, myocardial infarction, and ischemic stroke [6–8]. Although the underlying mechanisms are unclear, it is now nearly universally accepted that the therapeutic effects of transplanted MSCs on tissue repair is not only due to their differentiation potential into appropriate cell types that repairs and replaces the damaged tissues, but rather due to enhancing the function of resident cells via paracrine signaling actions [9]. There is accumulating evidence suggesting that MSCs synthesize and secrete multiple bioactive molecules such as angiogenic factors, growth factors and cytokines which have beneficial effects of endothelial cell behavior in vitro and induce angiogenic programing in vivo [10, 11].

In our previous study, we have shown that systemic transplantation of MSCs through caudal veins can accelerate reendothelialization and thereby prevents restenosis of vein grafts [12]. The transplanted cells could migrate to the inner walls of the vein graft, where they differentiate into functional endothelial cells, replace the damaged endothelium, and thus help prevent autologous vein graft restenosis. Recently, we found that, miRNA-126-3p, an EC-specific angiogenic miRNA, could improve cell function in human saphenous vein endothelial cells (HSVECs), other than in human saphenous vein smooth muscle cells (HSVSMCs) in vitro. Further research confirmed the results that therapeutic upregulation of miRNA-126-3p could repair dysfunctional endothelium and further prevent vein graft restenosis in a rat vein grafting model [13]. Therefore, we hypothesized that combined miRNA-126-3p and MSCs therapy may not only exert the advantage of MSC engraftment and subsequent transdifferentiation to replace damaged ECs, but also exhibit the benefit of MSC secretory function to repair neighboring resident endothelial tissues.

To date, no studies have explored the role of miRNA-126-3p and MSCs therapy in the field of vein graft restenosis. In this study, we transfected the miRNA-126-3p gene into human umbilical cord MSCs (hucMSCs) by lentiviral vector infection and investigated the effects of miRNA-126-3p overexpression on VEGF-induced endothelial cell proliferation, migration, and angiogenesis in vitro. We further investigated the partial downstream target genes and mechanisms involved in the promotive effects of EC activities. We also established an arterialized rat vein graft model and tested the therapeutic potential of miRNA-126-3p-hucMSC to accelerate the reendothelialization and inhibit neointimal hyperplasia in vivo. The aim of the present study was to investigate whether the combined miRNA-126-3p and stem cell-based therapy could produce synergistic effects and pose potential new therapeutic strategy for vein graft disease treatment.

## Materials and methods

### Isolation, characterization, and culture of hucMSCs and HUVECs

All experiment protocols were approved by the Ethical Committee of the Qilu Hospital of Shandong University (KYLL-2017-106) as we have described [14]. Human umbilical cords were collected from informed, consenting delivery woman and processed as we have previously described [14]. Human umbilical cord mesenchymal stem cells (hucMSCs) and human umbilical vein endothelial cells (HUVECs) were isolated, cultured, thawed, and expanded following the established method [15]. The hucMSCs were cultured in alpha-minimum essential medium (α-MEM) containing 10% fetal bovine serum (Gibco, USA) in a humidified atmosphere at 37 °C and 5% CO_2_. Different passages of hucMSCs were characterized by cell surface markers (CD29, CD34, CD44, CD45, CD90, CD105, CD166, and HLA-DR) using flow cytometric analysis, and multi-lineage differentiation potential were tested using osteogenic medium and adipogenic medium. The HUVECs were cultured in endothelial cell growth medium (ECM) supplemented with 5% fetal bovine serum in 5% CO_2_ at 37 °C. Endothelial phenotype was analyzed by CD31 and VWF immunofluorescence staining.

### Transfection of hucMSCs with miRNA-126-3p by lentiviral vector

The hucMSCs were transfected with miRNA-126-3p by lentiviral vector as previously described [16]. Briefly, a lentiviral vector expressing both the miRNA-126-3p and green fluorescent protein (GFP) was used for gene delivery. Primary hucMSCs were incubated with recombinant miRNA-126-3p-GFP or miRNA-126-3p NC-GFP lentiviral vectors at a multiplicity of infection (MOI) of 0, 25, 50, 75, and 100, respectively. The cells were infected with the lentivirus medium for 6 h and replaced with fresh medium for another 48 h. The green fluorescent protein signal was detected by a fluorescence microscope and gene transfection efficiency was verified by PCR.

### Co-culture of hucMSCs and HUVECs

HUVECs and hucMSCs were co-cultured using Transwells (0.4 μm pore size, Corning, USA) according to the manufacturer’s instructions. Briefly, HUVECs suspended in ECM stimulate with 100 ng/mL VEGF was added to the upper chamber with 0.4-μm diameter pores. Then, the transfected hucMSCs (miRNA-126-3p-hucMSCs or GFP-hucMSCs) were cultured with α-MEM in the lower chamber. After the 24 h incubation period, the HUVECs were collected for subsequent functional and mechanism analysis.

### Endothelial migration assay

Migration of co-cultured HUVECs was examined by scratching assay and Transwell analysis. For scratching assay, HUVECs co-cultured as aforementioned were harvested and seeded in 6-well plates. Scratching was mechanically made 6 h later using a 200-μl pipette tip. After 24 h, defect closing images were taken by using a microscopy and the migrated cells was counted for the cells in three field per well of each group. For Transwell analysis, co-cultured HUVECs were seeded to the upper chamber and the lower chamber was filled with ECM by using a 24-well Transwell cell culture chamber (8 μm pores). After 24 h, HUVECs on the upper surface were erased and migrated cells on the underside of chamber were fixed and stained with crystal violet. The transmembrane cells were imaged and counted using an inverted microscope in 5 randomly selected 200 fields per Transwell.

### Endothelial proliferation assay

Proliferation of co-cultured HUVECs was examined by EDU incorporation assay with an EDU imaging kit according to the manufacturer’s protocol. The co-cultured HUVECs in 24-well plates were preincubated with EDU-containing medium and fixed with 4% paraformaldehyde. The cells were then treated with TrionX-100 and EDU reaction buffer. Lastly, the nuclei were stained with DAPI. The proliferation rate was evaluated by the percentage of EDU stained cells divided by DAPI stained cells.

### In vitro angiogenesis assay

HUVECs were incubated in 24-well plates containing Coster Matrigel TM matrix (354234) under the same conditions as mentioned above. After cells were incubated in ECM for 24 h, the tube-like structures were formed and the tube morphology was analyzed under an inverted microscope. Image J was applied for quantifying the statistics of tube number and length.

### Extraction and identification of hucMSCs-exosomes

hucMSCs-exosomes were extracted from supernatants of cell cultures by ultracentrifugation method as we have described [14]. The morphology hucMSCs-exosomes were observed under a transmission electron microscope (Hitachi, Japan) by an experienced engineer. Western blot analysis was performed to identify the representative marker of exosomes, including CD63, HSP70, and TSG101 [17]. To explore whether hucMSCs-exosomes can be uptake by HUVECs, hucMSCs-exosomes were labeled with a fluorescent dye CM-Dil and incubated with HUVECs for 12 h at 37 °C. The cells were fixed in 4% paraformaldehyde and observed under the confocal laser microscopy (Zeiss, Germany).

### Surgical procedure and therapeutic interventions of rat vein grafting model

Male Wistar rats weighing 250–300 g purchased from the animal experimental center of Shandong University, with approval from the Institutional Animal Care and Use Committee at Qilu Hospital of Shandong University, were used in this experiment. The vein graft model and hucMSC transplantation were performed by using our previously described methods [18, 19]. Briefly, rats were randomly divided into 5 groups as follows: [1] sham group, [2] vein graft group, [3] vein graft+hucMSCs group (hucMSCs group), [4] vein graft+GFP-hucMSCs group (GFP-hucMSCs group), and [5] vein graft+miRNA-126-3p-hucMSCs group (miRNA-126-3p-hucMSCs group). End-to-end anastomosis using “cuff technology” was performed to establish arteriovenous bypass grafting model in the vein graft group and all three hucMSC transplantation groups. All three cell transplantation groups were injected with 200 μL PBS with 2 × 10 [6] native hucMSCs or genetically engineered hucMSCs (GFP-hucMSCs or miRNA-126-3p-hucMSCs) via the tail vein 24 h after vein grafting. The vein graft group was treated with an equal volume of PBS. The sham group underwent neither vein grafting nor hucMSC transplantation except that the external jugular vein was carefully divided.

### Homing of transplanted MSCs to vein grafts

Vascular ultrasound examinations were performed on all rats before and after operation, luminal diameter and blood flow were measured using a small animal ultrasound scanner with a linear array transducer at 4 weeks after operation. CM-Dil-labeled hucMSCs observed by a fluorescence microscope (Olympus, Japan) was used to examine the homing of transplanted hucMSCs to the site of the vein grafts 3 days after transplantation. Endothelial regeneration was evaluated by CD34 immunofluorescence 14 days after vein grafting as we have previously described [13]. All the remaining animals were then humanely killed and the vein grafts samples were harvested. The therapeutic effects of hucMSCs were evaluated by subsequent histological and immunohistochemical examination.

### Histological and immunohistochemical examination of vein grafts

For histological study, the grafted veins were collected, fixed, and embedded. Paraffin-embedded sections (5 μm) were processed for morphometrical analyses after hematoxylin-eosin (HE) staining and Masson’s trichrome staining [13]. The thickness of the intima in the vein grafts were used to estimate neointimal hyperplasia in different groups. Immunohistochemistry was performed by using a detection kits according to the manufacturer’s recommendation. Vein graft sections were incubated with markers for cell proliferation (Ki67), macrophage infiltration (CD68), cytokines infiltration (TNF-α), and counterstained with hematoxylin and viewed under a microscope [13].

### Western blot analysis

Western blot analysis was performed according to standard protocol as we have previously described [13]. In brief, total proteins were extracted from cells by treating with RIPA buffer. The extracts were quantified using a BCA protein assay kit and separated by SDS-PAGE and electroblotted onto a PVDF membrane. After blocking, the protein blots were incubated with the following antibodies: PIK3R2, SPRED-1, AKT, p-AK, ERK1/2, p-ERK1/2, and GAPDH, followed by incubation with secondary antibodies. Immunoreactive bands were visualized by using an enhanced chemiluminescence kit.

### RNA extraction and real-time quantitative polymerase chain reaction (PCR)

For analysis of miRNA-126-3p expression, total RNA was extracted using RNA Isolation Kit and reverse-transcribed using All-in-One TM miRNA First-Strand cDNA Synthesis Kit following the manufacturer’s instructions. For analysis of SPRED-1 and PIK3R2 expression, a ReverTra Ace q-PCR RT Kit was used to synthesize the first-strand. PCR was performed in triplicate using a Real-time Detection System (BioRad, USA) and gene transcripts were analyzed using the delta-delta CT method.

### Statistical analysis

The experiments were performed 3 times and the values were expressed as mean ± standard deviation. The results were assessed statistically using Student’s *t* test between two groups, or analyzed by one-way analysis of variance between three or more groups. Statistical analysis was performed by using GraphPad Prism Software. A value of *P* < 0.05 was considered statistically significant.

## Results

### Characterization of hucMSCs and HUVECs

**Fig. 1:**
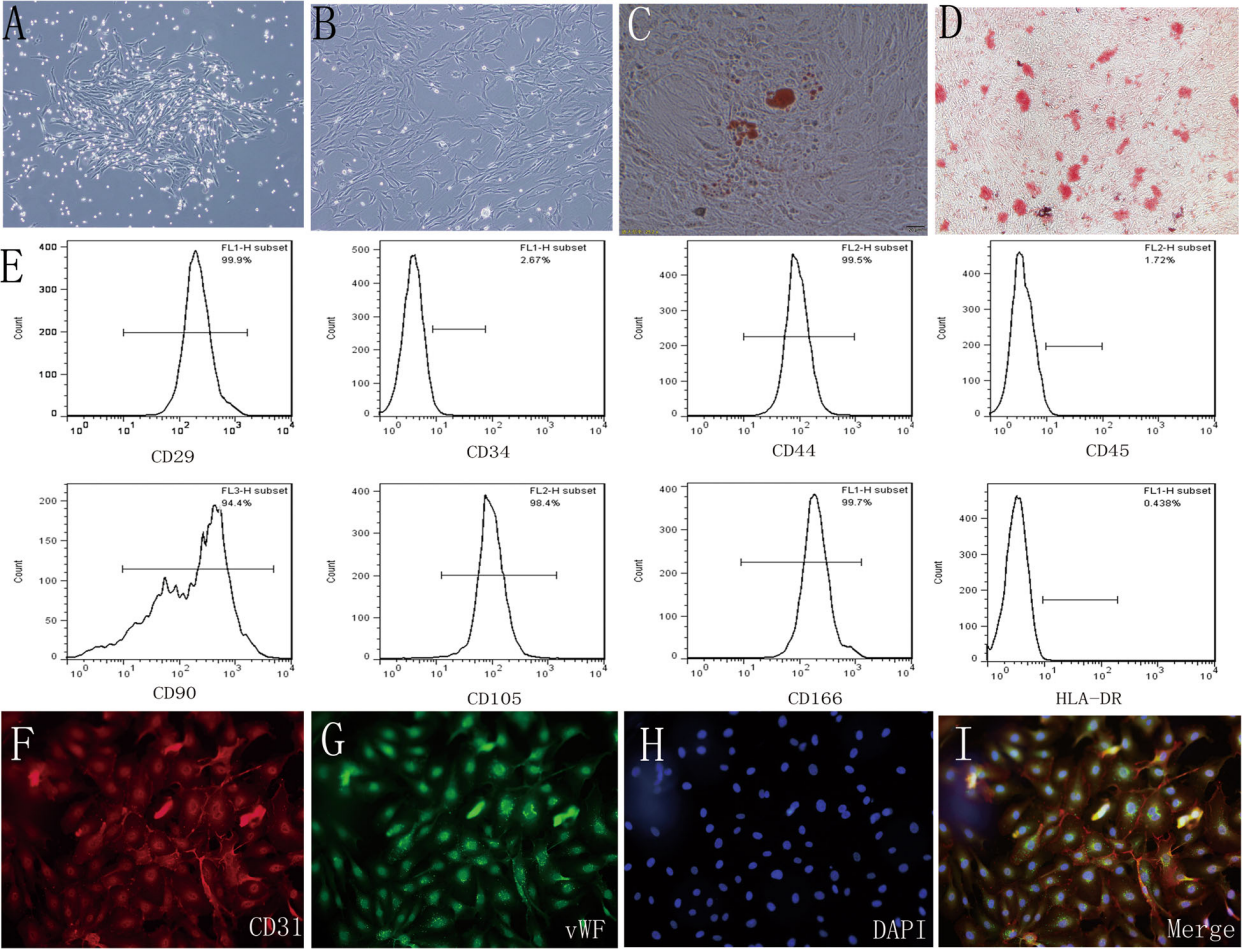
Characterization of hucMSCs and HUVECs. **a** hucMSCs formed cell colony 3 days following isolation. **b** hucMSCs exhibited a fibroblast-like shape at passage 3. **c** hucMSCs were stained with Oil Red O to assess their adipogenic ability. **d** hucMSCs were stained with Alizarin Red to assess their osteogenic ability. **e** Flow cytometry analysis suggested that cultivated cells expressed strongly expressed CD29, CD44, CD90, CD105, and CD166 and HLA-DR but did not express CD34 and CD45. **f** Immunostaining of hucMSCs showing CD31 (red) and vWF (green) **g** staining. **h** Cell nuclei were stained with DAPI. **i** The merged images

As shown in Fig. 1a, cell colonies were formed in the plastic tissue culture dishes after 3 days following isolation, and approximately all hucMSCs at passage 3 were flattened fibroblast-like in shape (Fig. 1b). The results of Oil Red O staining and Alizarin Red staining indicating that the hucMSCs could be induced to differentiate into adipocytes and osteoblasts (Fig. 1c, d). hucMSCs strongly expressed stem cell surface markers CD29, CD44, CD90, CD105, CD166, and HLA-DR but were negative for CD34 and CD45, as shown by flow cytometry analysis (Fig. 1e). We isolated primary HUVECs and identified them by using CD31 and vWF double immunofluorescence, which confirmed that 95% of cells were double positive (Fig. 1f, i). All these results showed that the purity of cultured cells can satisfy the experiment requirements.

### Successful transfection of miRNA-126-3p into hucMSCs

**Fig. 2:**
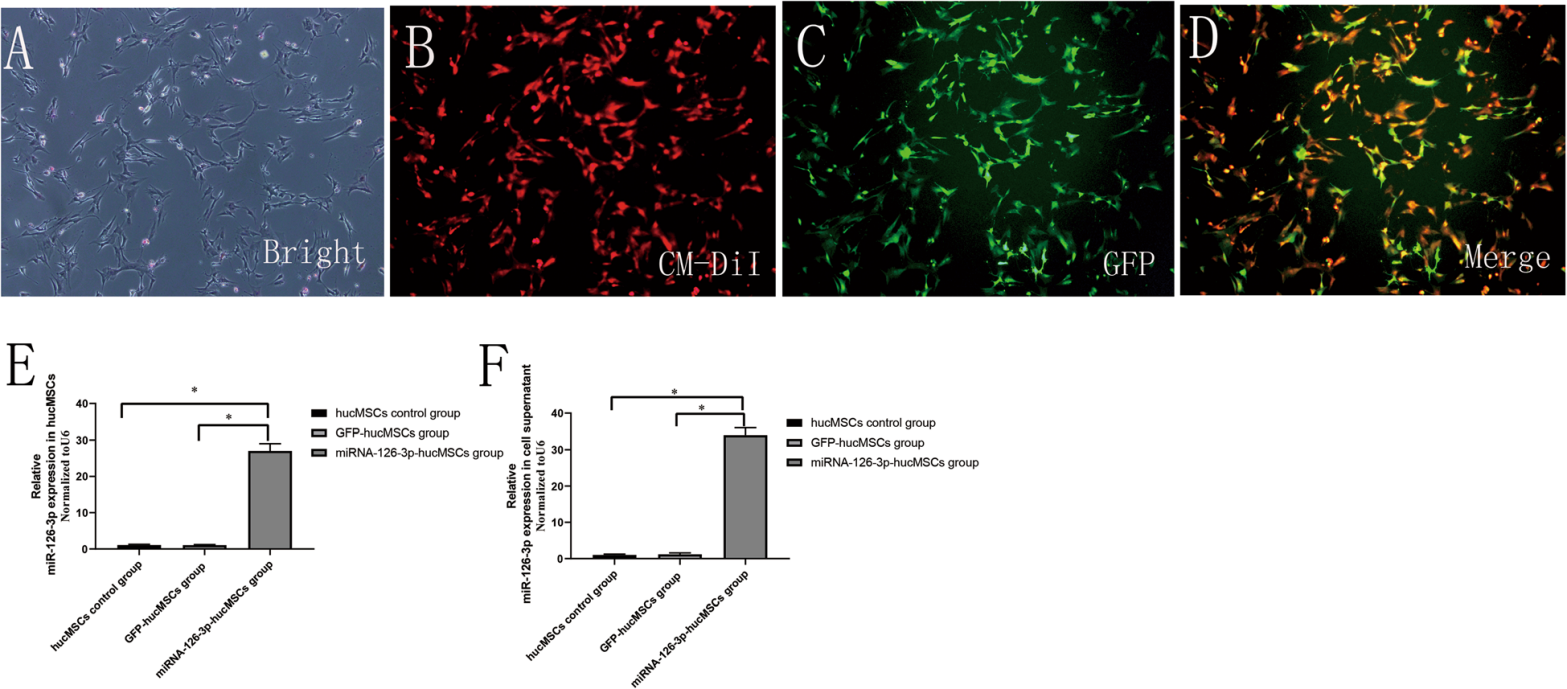
miRNA-126-3p transduction by lentiviral vectors. **a** Primary hucMSCs were transfected with lentiviral vectors carrying miRNA-126-3p-GFP. **b** CM-DiI-labeled MSCs showed red fluorescence under fluorescence microscope. **c** GFP image of hucMSCs at 72 h after transduction by lentiviral vectors. **d** The merged images. **e** The transfection efficiency of miRNA-126-3p was evaluated by RT-PCR. **f** Secretion of miRNA-126-3p in the cell culture supernatants of hucMSCs and GFP-hucMSCs and miRNA-126-3p-hucMSCs

Almost all the hucMSCs exposed to CM-Dil showed red fluorescence under fluorescence microscope (Fig. 2a, b). Primary hucMSCs were transfected with lentiviral vectors carrying either miRNA-126-3p-GFP or miRNA-126-3p NC-GFP at a MOI of 0, 25, 50, 75, and 100. Almost all of the cultured cells were GFP positive when the MOI = 75; hence, this MOI value was initially chosen for the optimum concentration in the following experiments (Fig. 2c, d). Real-time quantitative PCR data indicated that the expression of miRNA-126-3p in the miRNA-126-3p-hucMSCs group was 27-fold higher than that in the GFP-hucMSCs group (*P* < 0.01), and 26-fold higher in expression than the hucMSCs control group (*P* < 0.01, Fig. 1e). But more importantly, the levels of miRNA-126-3p expression in cell culture supernatants of the miRNA-126-3p-hucMSCs group was 34-fold higher than that from the GFP-hucMSCs group and hucMSCs control group as determined by PCR (*P* both < 0.01, Fig. 2f).

### Overexpression of miRNA-126-3p in hucMSCs regulates HUVECs proliferation, migration, and tube formation

**Fig. 3:**
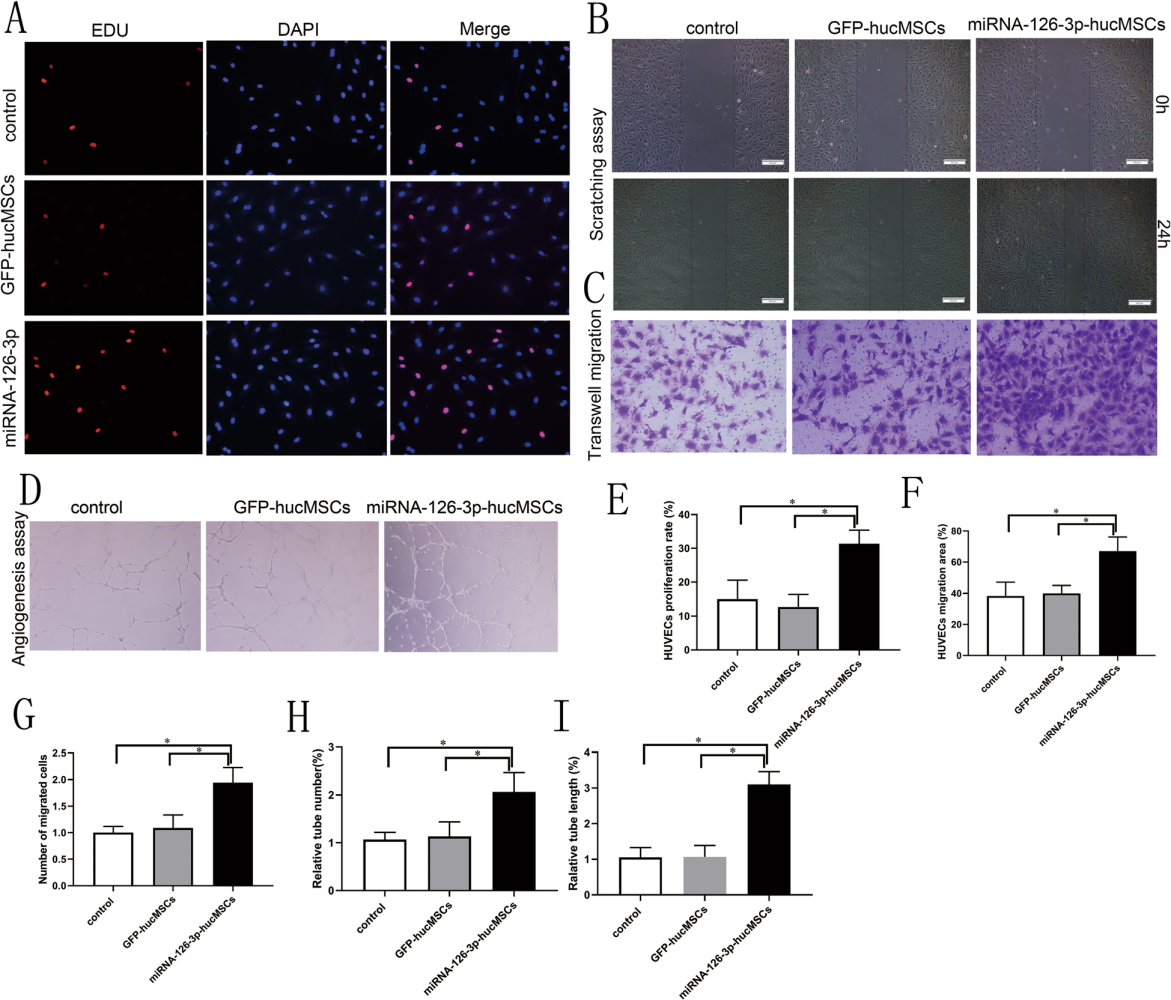
Overexpression of miRNA-126-3p in hucMSCs regulates HUVECs proliferation, migration, and tube formation. **a** The images of the EDU incorporation assay of HUVECs co-cultured with hucMSCs. **b** The images of cell scratching assay of HUVECs co-cultured with hucMSCs. **c** The images of cell Transwell migration assay of HUVECs co-cultured with hucMSCs. **d** The images of the tube formation assay of HUVECs co-cultured with hucMSCs. **e** Quantification of the proliferation rates of HUVECs. **f** Quantification of the migration area of HUVECs. **g** Quantification of the migrated cells numbers of HUVECs. Quantification of the tubes numbers (**h**) and tube length (**i**) of HUVECs

Due to miRNA-126-3p being an endothelial special miRNA, we analyzed the miRNA-126-3p-hucMSC function in vascular endothelial cells by using a series of co-culture experiments. We originally used the EDU incorporation to examine whether the miRNA-126-3p overexpression in hucMSCs could affect the HUVECs proliferation stimulated by VEGF. The EDU incorporation analysis indicated that overexpression of miRNA-126-3p in hucMSCs markedly increased the percentage of EDU-positive stained HUVECs compared to the GFP-hucMSCs group or hucMSCs control group, in which no difference was observed between the GFP-hucMSCs group and hucMSCs control group (Fig. 3a, e).

We next performed cell scratching assay to estimate the influence of miRNA-126-3p-hucMSCs in VEGF-induced HUVECs migration. The result indicated that overexpression of miRNA-126-3p in hucMSCs significantly increased the size of the migration area of HUVECs (Fig. 3b, f). We further used the Transwell migration assay to validate this result. An obvious increase of migrated cells was observed in the miRNA-126-3p-hucMSCs group compared with the control group (Fig. 3c, g).

Lastly, we performed in vitro angiogenesis assay to estimate the influence of miRNA-126-3p-hucMSCs in VEGF-induced tube formation of HUVECs. The tube formation assay showed that miRNA-126-3p transfected hucMSCs profoundly increased the tube numbers and tube length of co-cultured HUVECs compared with scrambled miRNA transfected cells and the control group (Fig. 3d, h, i). Taken together, our co-culture experiments revealed that overexpression of miRNA-126-3p in hucMSCs could activate an angiogenic program in co-cultured HUVECs through a paracrine pathway, suggesting proangiogenic potential in vascular reendothelialization.

### Overexpression of miRNA-126-3p in hucMSCs regulates HUVECs proliferation, migration, and tube formation through exosome-mediated mechanisms

**Fig. 4:**
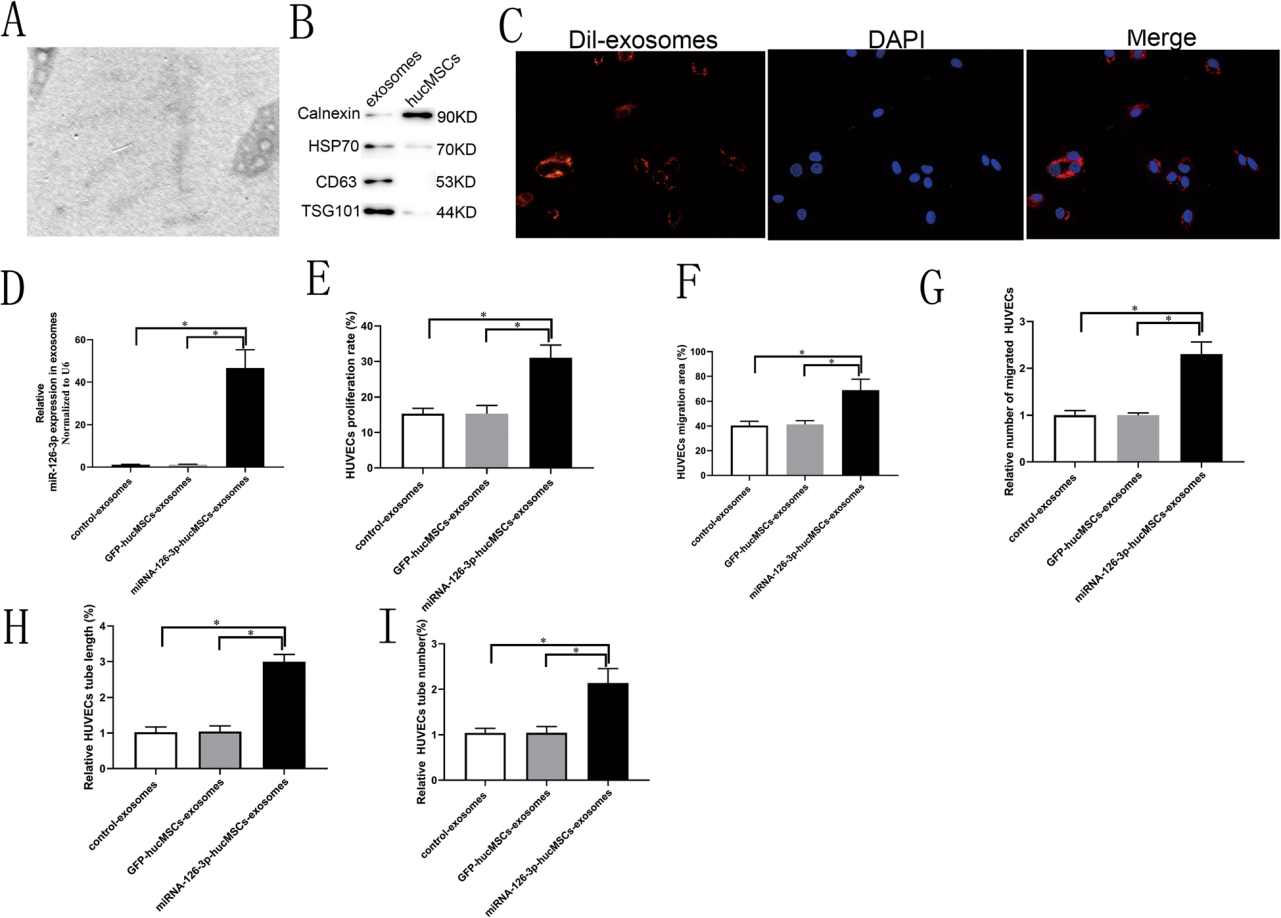
The role and mechanisms of miRNA-126-3p-hucMSCs-exosomes in the promotion of HUVECs proliferation, migration, and tube formation. **a** The morphology of hucMSCs-exosomes. **b** Western blotting analysis of exosome markers. **c** DiI-labeled hucMSCs-exosomes (red) could be internalized by DAPI-labeled HUVECs (blue). **d** The expression of miRNA-126-3p in exosomes derived from hucMSCs and GFP-hucMSCs and miRNA-126-3p-hucMSCs. **e** Quantification of the proliferation rates of HUVECs. **f** Quantification of the migration area of HUVECs. **g** Quantification of the migrated cell numbers of HUVECs. Quantification of the **h** tube length and tubes numbers (**i**) of HUVECs

It was shown that miRNA could be transferred from cell to cell via exosomes. In our previous study, we have found that, hucMSCs-exosomes can affect HUVECs function. In this study, we separated hucMSCs-exosomes in cell culture supernatants, visualized the morphology of exosomes under a transmission electron microscopy (Fig. 4a), and identified its specific exosome markers by calnexin, HSP70, CD63, and TSG101 western blot analysis (Fig. 4b). In our in vitro tracking experiment, we found that CM-Dil-labeled hucMSCs-exosomes can be internalized into endothelial cells (Fig. 4c). We observed that the levels of miRNA-126-3p expression in exosomes derived from miRNA-126-3p-hucMSCs (miRNA-126-3p-hucMSCs-exosomes) were 45-fold higher than that from GFP-hucMSCs (GFP-hucMSCs-exosomes) and hucMSCs as determined by PCR (*P* both < 0.01, Fig. 4d). The results provided biological basis for the potential functional influence of miRNA-126-3p-hucMSCs-exosomes on endothelial cells.

In order to examine the role of miRNA-126-3p-hucMSCs-exosomes in VEGF-induced HUVECs proliferative and migratory and angiogenic activities, HUVECs were given 50 μg/ml exosomes derived from control hucMSCs, GFP-hucMSCs, and miRNA-126-3p-hucMSCs, respectively. Using the function assays, we found that miRNA-126-3p-hucMSCs-exosomes not only strengthen significantly the proliferative and migratory capacities of HUVECs (Fig. 4e–g), but also increased the formation of tube-like structures compared to hucMSCs-exosomes and GFP-hucMSCs-exosomes in vitro (Fig. 4h, i). As expected, no difference was observed between the hucMSCs-exosomes and GFP-hucMSCs-exosomes. We can conclude that overexpression of miRNA-126-3p in hucMSCs change HUVECs function was mediated exosome mechanisms, at least in part.

### Overexpression of miRNA-126-3p in hucMSCs regulates ERK1/2 and AKT signaling through targeting SPRED-1 and PIK3R2 genes in HUVECs

**Fig. 5:**
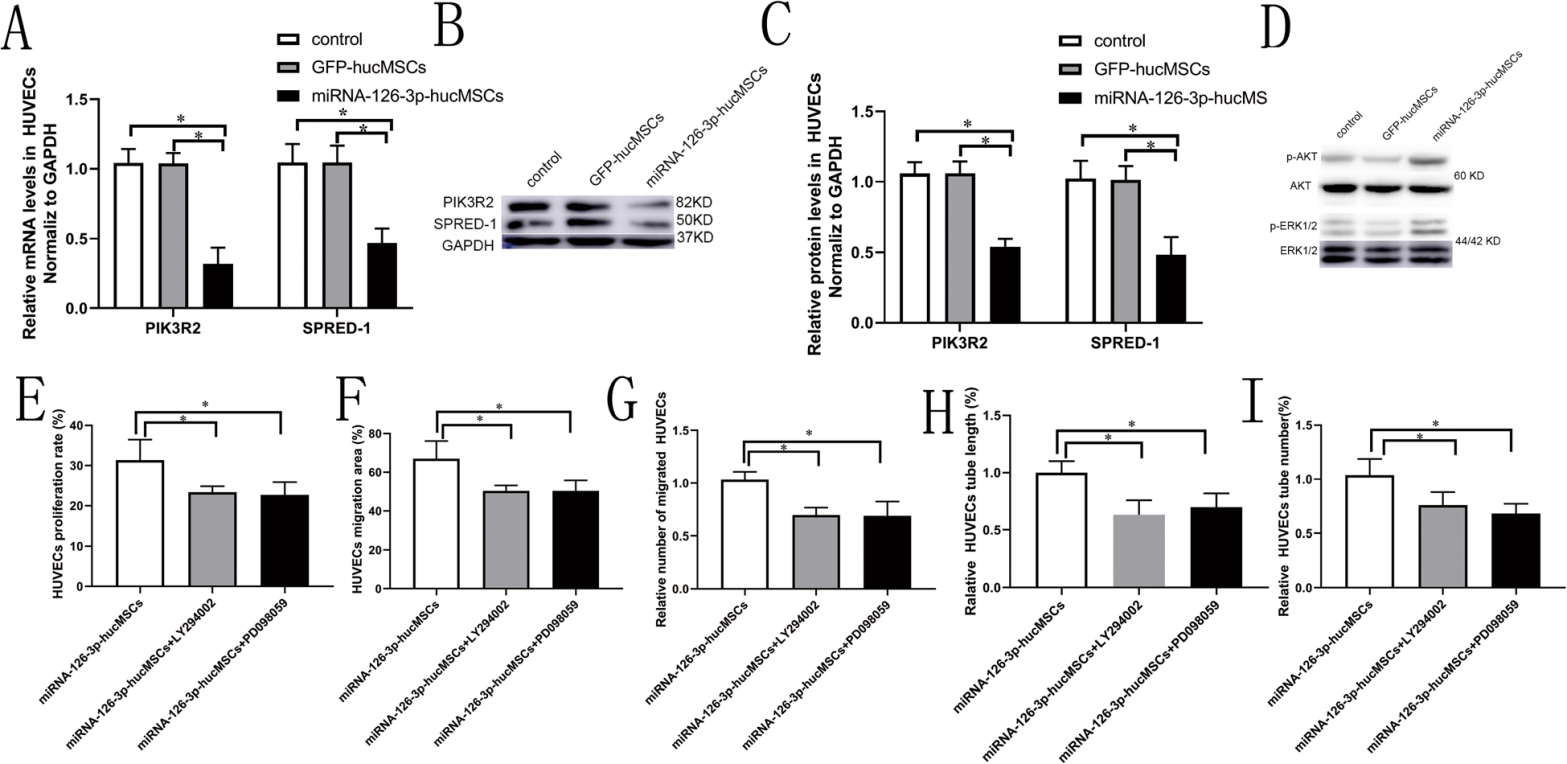
**a** SPRED-1and PIK3R2 mRNA levels were measured in HUVECs co-cultured with hucMSCs and GFP-hucMSCs and miRNA-126-3p-hucMSCs. **b** Western blots of PIK3R2 and SPRED-1 protein expression in HUVECs co-cultured with hucMSCs and GFP-hucMSCs and miRNA-126-3p-hucMSCs. **c** SPRED-1 and PIK3R2 protein levels were higher in the miRNA-126-3p-hucMSCs group than in the hucMSCs or GFP-hucMSCs group. **d** Representative western blots of AKT phosphorylation and ERK1/2 phosphorylation in HUVECs co-cultured with hucMSCs and GFP-hucMSCs and miRNA-126-3p-hucMSCs. **e** Quantification of the proliferation rates of HUVECs. **f** Quantification of the migration area of HUVECs. **g** Quantification of the migrated cell numbers of HUVECs. **i** Quantification of the tube length and tubes numbers of HUVECs

Our initial investigation has demonstrated that SPRED-1 and PIK3R2 are targets of miRNA-126-3p to critically influence angiogenic activities in endothelial cells [13]. In this study, we further performed co-culture experiments to detect downstream target genes of miRNA-126-3p in HUVECs. As illustrated in Fig. 5a–c, compared with the GFP-hucMSCs group and hucMSCs control group, SPRED-1 and PIK3R2 protein and mRNA expression was significantly decreased in the miRNA-126-3p-MSCs group. Furthermore, phosphorylation levels of AKT and ERK1/2 was markedly increased in the miRNA-126-3p-hucMSCs group (Fig. 5d). No significant changes in the expression of SPRED-1 and PIK3R2 genes and phosphorylated ERK1/2 and AKT in the GFP-hucMSCs group and hucMSCs control group. We also added AKT inhibitor LY294002 and ERK1/2 inhibitor PD098059 in the co-culture systems. As expected, AKT and ERK1/2 inhibitors almost completely blocked the promotion effects of miRNA-126-3p-hucMSCs in HUVECs proliferation, migration, and tube formation (Fig. 5e–i). The results were consistent with our previous study that miRNA-126-3p promoted human saphenous vein endothelial cells function through the enhancing of ERK1/2 and AKT signaling pathways and the downregulation of SPRED-1 and PIK3R2 gene expression [13].

### Enhanced reendothelialization of vein graft by transplantation of hucMSCs overexpressing miRNA-126-3p in vivo

**Fig. 6:**
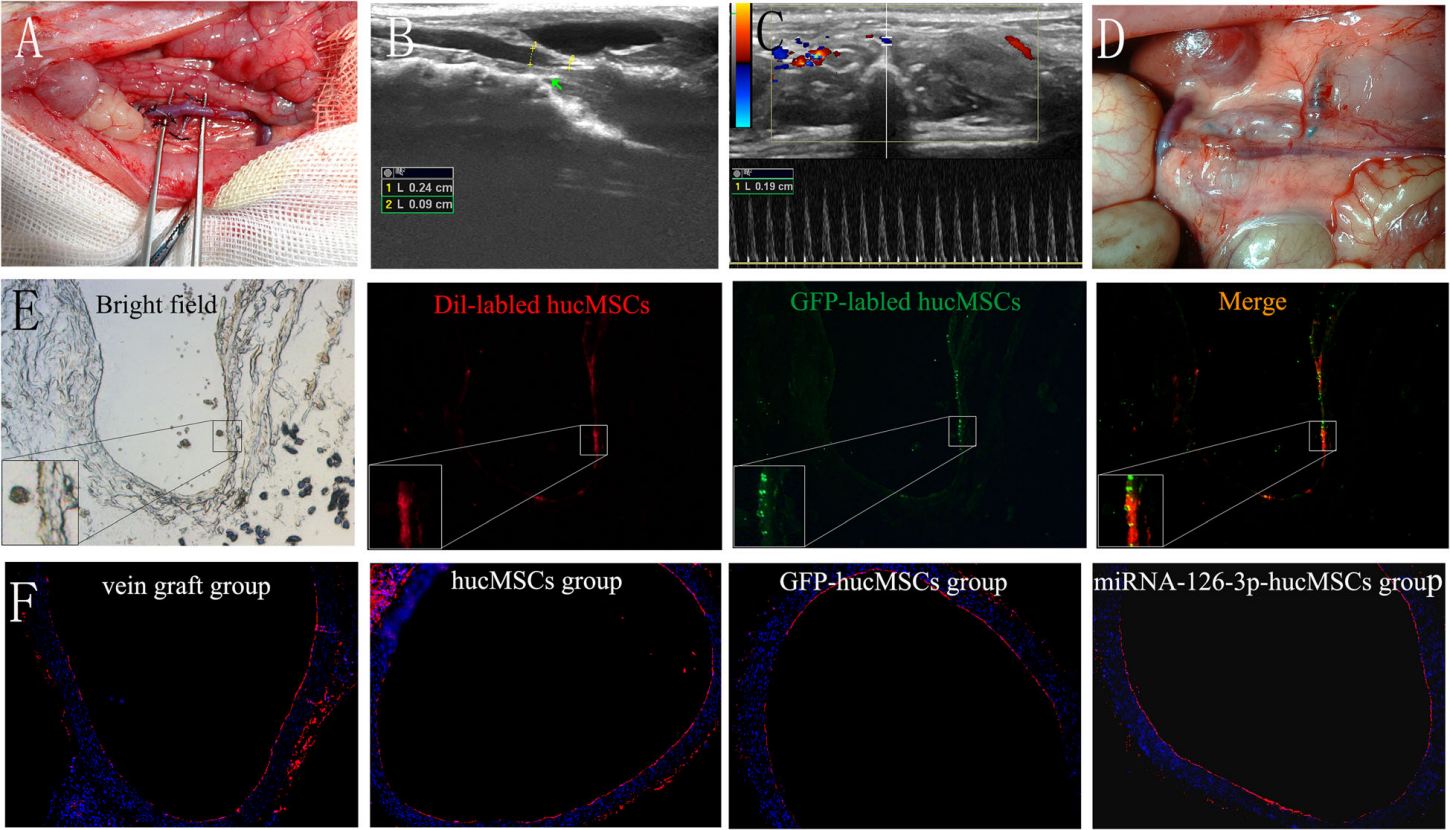
Treatment with hucMSCs overexpressing miRNA-126-3p accelerated endothelialization of the vein grafts in an arterialized rat model. Rat vein graft model: external jugular vein into infrarenal aorta (**a**). The luminal diameter (**b**) and peak systolic velocity (**c**) were measured by serial ultrasound studies during the whole study period. **d** Representative gross morphology of vein grafts at 4 weeks after transplantation. **e** Representative images of hucMSCs recruitment in the vein grafts, hucMSCs were predominantly found at the intraluminal site of the blood vessel as indicated by CM-Dil and GFP fluorescence. **f** Vein grafts harvested 2 weeks after hucMSC transplantation were analyzed by CD34 immunohistochemical staining to assess vascular reendothelialization in each group

In our previous study, we have proved engrafted MSCs present in vein graft walls could differentiate into functional endothelial cells and accelerate reendothelialization [12]. We also demonstrated that local upregulation of miRNA-126-3p in vein graft walls after venous implantation could accelerate reendothelialization and thereby attenuates neointimal hyperplasia [13]. To assess whether miRNA-126-3p overexpression in hucMSCs can further accelerate reendothelialization process in vivo, an animal experiment was carried out on a rat vein grafting model (Fig. 6a–d). The homing of transplanted hucMSCs was tracked by CM-Dil labeling 3 days after the transplantation. Immunofluorescence studies showed that CM-Dil-positive cells localized primarily in the inner walls of vein grafts (Fig. 6e). The CD34 immunohistochemical staining of the vein graft luminal surface 2 weeks after hucMSC transplantation is shown in Fig. 5f. Endothelialization of the vein grafts were significantly enhanced in the hucMSCs group, GFP-hucMSCs group, and miRNA-126-3p-hucMSCs group compared with the vein graft group. However, hucMSCs modified with miRNA-126-3p had a higher endothelialization of the vein grafts when compared with the GFP-hucMSCs or the hucMSCs control groups.

### Enhanced inhibition of neointimal hyperplasia by transplantation of hucMSCs overexpressing miRNA-126-3p in vivo

**Fig. 7:**
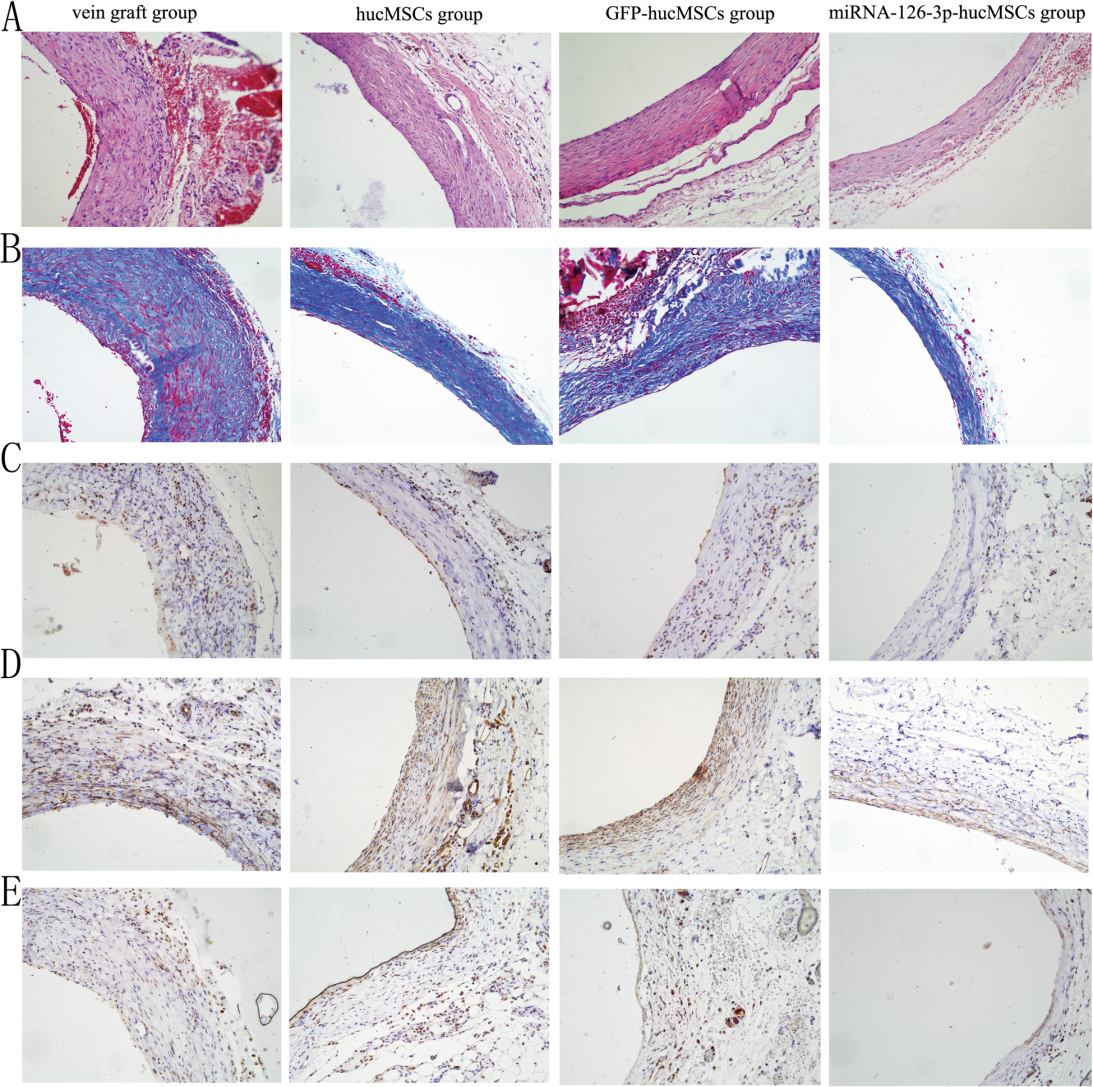
Treatment with hucMSCs overexpressing miRNA-126-3p attenuated vein graft intimal hyperplasia and vascular inflammation in a rat model. **a** Representative images of the vein graft stained with HE. **b** Representative images of the vein graft stained with Masson’s trichrome. **c** Representative images of the vein graft stained with Ki67. **d** Representative images of the vein graft stained with TNF-α. **e** Representative sections of the vein graft stained with CD68

We next tested if hucMSC with miRNA-126-3p overexpression could achieve better efficiency in attenuating neointimal hyperplasia in these vein grafting models. The effect of hucMSC transplantation on vascular lesion development and extracellular matrix synthesis in vein grafts are shown in Fig. 7a by HE staining and Fig. 7b by Masson’s trichrome staining. All three cell transplantation groups had significantly decreased neointima hyperplasia compared with the vein graft group. However, the intimal thickness decreased more obviously in the miRNA-126-3p-hucMSCs group than the other two cell transplantation groups. No significant differences were found between hucMSCs group and GFP-hucMSCs group.

We performed Ki67 immunohistochemical staining to examine cell proliferation in the neointima. Compared with the hucMSCs group and GFP-hucMSCs group, it could induce an obvious reduction in the Ki67-positive cells in the vascular wall after treatment with miRNA-126-3p-hucMSC (Fig. 7c). The damage of endothelial cells triggered inflammatory cascade has been documented to contribute to neointimal hyperplasia in vein grafts [20]. In this study, the expression of inflammatory cells and cytokines in grafted veins were assessed by TNF-α and CD68 immunohistochemistry. As shown in Fig. 7d and e, positive staining for TNF-α and CD68 were found in the inner, mid, and outer vascular layers of grafted veins; the results show that delivery of miRNA-126-3p-hucMSC decreases the expression of TNF-α protein and CD68-positive cells. These data further confirm that intravenous implantation of miRNA-126-3p-hucMSCs appeared to have better protective roles towards vein graft restenosis as compared to treatment using hucMSCs or GFP-hucMSCs.

## Discussion

Up to 50% of the autogenous vein will be restenosis or occluded during the first 10 years after CABG, leading to recurrent symptoms, myocardial infarction, or reoperative revascularization [2]. Currently, there is no effective treatment strategy for vein graft restenosis. It is widely accepted that neointimal hyperplasia is the leading cause of vein graft failure, and selectively accelerate dysfunction endothelial recovery after vein grafting was shown to improve their long-term patency and therefore benefit the therapeutic effect of CABG [21].

**Fig. 8:**
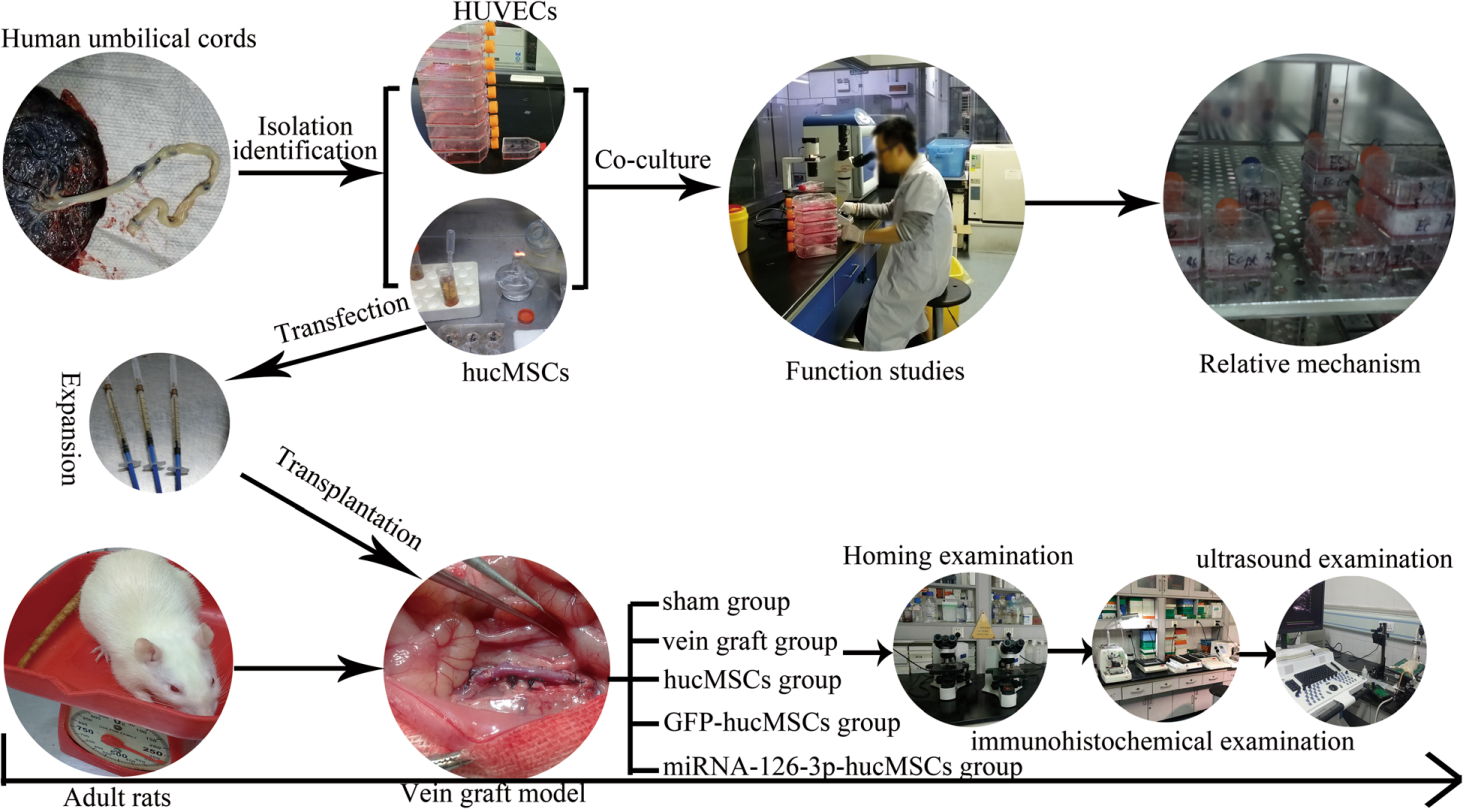
Schematic representation of in vivo and vitro experimental design and relevant morphology

In this study, using a series of co-culture experiments, we found that combined miRNA-126-3p and human umbilical cord mesenchymal stem cell therapy could strengthen endothelial cells proliferation, migration, and tube formation ability through exosome-mediated mechanisms in vivo. Besides, we also find that SPRED-1 and PIK3R2 target gene and ERK1/2 and AKT signaling pathway participated in the process. We verified that intravenous implantation of miRNA-126-3p-hucMSCs appeared to have higher reendothelialization percentage and lower neointima hyperplasia towards vein graft restenosis as compared to treatment using hucMSCs or GFP-hucMSCs in a rat vein arterialization model. The in vivo and vitro experimental design and relevant morphology were summarized in Fig. 8. There were studies the suggested rat vein grafts generate a completely neointimal formation after 4 weeks [22, 23]. Due to the limited of rat vein graft model, the long-term effects of miRNA-126-3p-hucMSCs against restenosis of vein grafts were not investigated in the current study. All our results have demonstrated that hucMSC-based miRNA-126-3p gene therapy is a promising approach to prevent vein grafts remodeling in the formation of vascular restenosis.

Numerous studies have shown that MSCs have the clinical therapeutic value for the treatment of vascular diseases because of their transdifferentiation and paracrine characteristics [9]. In our previous study, we have found that systemic transplantation of bone marrow-derived mesenchymal stem cells (BMSCs) could migrate to the inner walls of the vein graft, where they differentiate into functional endothelial cells, replace the damaged endothelium, and thus prevent intimal hyperplasia by accelerating reendothelialization [12]. Though we have demonstrated BMSCs implantation plays a significant role in vein graft neointimal hyperplasia and reendothelialization, we selected hucMSCs as vector cells for this study [12, 19]. Compared with BMSCs, hucMSCs have many advantages for the further clinic application, such as abundant source, ease of harvesting, painless accession, no donor site morbidity, low immunogenicity, and faster self-renewal in vitro [24].

It is widely accepted that miRNA-126-3p is one of the most important endothelial cell-restricted miRNAs that regulate vascular integrity and developmental angiogenesis [25, 26]. The proangiogenic effect of miRNA-126-3p has been attributed to target genes SPRED-1and PIK3R2, which are the negative regulators VEGF signaling pathway, leading to increased vasculogenesis-related in vitro parameter, such as proliferation, migration, and tube formation. In one of our previous study, we have found that miRNA-126-3p plays a protective role in vein graft disease [13]. Local delivery of miRNA-126-3p by agomir inhabited intimal hyperplasia in human and rat vein grafts. The mechanisms may involve regulating of the VEGF /ERK1/2 and AKT pathways via the target genes of SPRED-1 and PIK3R2 [13]. Therefore, miRNA-126-3p is a promising candidate for gene modifications. In this study, we provided enough evidence to conclude that combined miRNA-126-3p and hucMSCs therapy can absolutely give play to their respective superiorities.

Many studies have proved that exosomes secreted by MSCs may be a key ingredient of paracrine mechanism to spread its repair function, exosomes can be served as a translocator (miRNAs, lncRNAs, and proteins) that contributes to intercellular communication [27, 28]. Our previous studies have found that exosome cargo may account for the vascular protective effect of hucMSCs against vein graft failure [14]. To confirm whether the miRNA-126-3p-hucMSC transplantation on endothelial function is by a paracrine pathway through the exosome mechanisms, we separated miRNA-126-3p-hucMSCs-exosomes in cell culture supernatants and evaluated its effects on endothelial cells by integrated experiments and co-culture experiments in this study. The results showed that overexpression of miRNA-126-3p in hucMSCs enhances endothelial function through exosome-mediated mechanisms, at least in part.

## Conclusions

In conclusion, the present study is the first published report to explore the effect of combined gene and stem cell-based therapy on prevention of neointimal formation associated with vein graft failure. We have demonstrated, for the first time, that miRNA-126-3p overexpression in hucMSCs enhances endothelial function through paracrine mechanism in vitro and thereby accelerate reendothelialization and attenuates vein grafts neointima hyperplasia in rats. These novel findings may have great clinical implications for the prevention of vein graft stenosis after CABG.

## Data Availability

Not applicable.

## Abbreviations

MSC: Mesenchymal stem cell
hucMSCs: Human umbilical cord MSCs
hucMSCs-exosomes: Exosomes derived from human umbilical cord MSCs
TEM: Transmission electron microscopy
VEGF: Vascular endothelial growth factor
CABG: Coronary artery bypass grafting
HUVEC: Human umbilical vein endothelial cell
VWF: Von Willebrand factor
PBS: Phosphate-buffered saline
HE: Hematoxylin and eosin
GFP: Green fluorescent protein
MOI: Multiplicity of infection
α-MEM: Alpha-minimum essential medium
HSVECs: Human saphenous vein endothelial cells
HSVSMCs: Human saphenous vein smooth muscle cells
